# Metabolic Syndrome Is Associated With Advanced Liver Fibrosis Among Pediatric Patients With Non-alcoholic Fatty Liver Disease

**DOI:** 10.3389/fped.2019.00491

**Published:** 2019-11-26

**Authors:** Yi-Wen Ting, Sui-Weng Wong, Azriyanti Anuar Zaini, Rosmawati Mohamed, Muhammad Yazid Jalaludin

**Affiliations:** ^1^Faculty of Medicine, University of Malaya, Kuala Lumpur, Malaysia; ^2^Endocrinology Unit, Department of Pediatrics, Faculty of Medicine, University of Malaya, Kuala Lumpur, Malaysia; ^3^Gastroenterology and Hepatology Unit, Department of Medicine, Faculty of Medicine, University of Malaya, Kuala Lumpur, Malaysia

**Keywords:** NAFLD, liver fibrosis, obesity, waist circumference, insulin resistance, transient elastography

## Abstract

**Background:** Non-alcoholic fatty liver disease (NAFLD) among children is a growing concern with potential significant outcome. This study aims to investigate the relationship between hepatic steatosis, metabolic syndrome, and liver fibrosis among children with obesity and diabetes mellitus.

**Methodology:** Children aged 6–18 years old were recruited from pediatric obesity and diabetes clinic in University Malaya Medical Center (UMMC) between year 2016 and 2019. Data on basic demographics, anthropometric measurements and clinical components of metabolic syndrome were collected. Transient elastography was performed with hepatic steatosis and liver fibrosis assessed by controlled attenuation parameter (CAP) and liver stiffness measurement (LSM) respectively. Mild, moderate and severe steatosis were defined as >248, >268, and >280 dB/m respectively, and LSM above 7.0 kPa for fibrosis stage F ≥ 2, 8.7 kPa for F ≥ 3, and 10.3 kPa for F4 (cirrhosis).

**Results:** A total of 57 children (60% male) with median age of 13 years old were recruited. Fifty (87.7%) of the children are obese and 27 (54%) out of 50 are morbidly obese. Among 44 (77.2%) patients with steatosis, 40 (70.2%) had severe steatosis and 18 (40.9%) had developed liver fibrosis of stage 2 and above. Advanced fibrosis or cirrhosis was detected in 8 (18.2%) children with presence of steatosis. Twenty-three out of 57 (40.4%) was diagnosed with metabolic syndrome. Fibrosis is three times more likely to occur in the presence of metabolic syndrome (OR = 3.545, 95% CI: 1.135–11.075, *p* = 0.026). Waist circumference is a significant predictor of fibrosis after multiple regression analysis.

**Conclusion:** Obese children with metabolic syndrome are more likely to have advanced liver fibrosis compared to those without metabolic syndrome. Waist circumference predicts development of liver fibrosis.

## Introduction

Non-alcoholic fatty liver disease (NAFLD) among pediatric population is increasing globally, and is currently one of the most common causes of chronic liver disease among young people in the developed world (1). According to a recent meta-analysis, prevalence of NAFLD in children from general population studies is reported to be around 7.6% and 34.2% in studies based on children's obesity clinics (2). NAFLD represents a spectrum that ranges from having fat alone without any inflammation or any evidence of liver cell injury (hepatocyte ballooning), referred to as steatosis or non-alcoholic fatty liver (NAFL), to non-alcoholic steatohepatitis (NASH), which is characterized by fat infiltration in the liver, lobular inflammation, and evidence of hepatocyte ballooning. NAFL is not benign as previously thought, with the possibility of progression to NASH, leading to fibrosis and ultimately liver cirrhosis and hepatocellular carcinoma (HCC) (3).

Metabolic syndrome describes a constellation of cardiovascular risk factors such as central adiposity, impaired glucose metabolism, hypertension, and dyslipidemia. Obesity and metabolic syndrome are closely related to NAFLD, predisposing patients to increased risk of cardiovascular events, other malignancies and mortality (4). In a 39 years follow-up study involving 44,248 male adolescents, overweight in late adolescence is found to be a significant predictor of severe liver disease later in life (5). Therefore, it is imperative to stratify patients according to their future risk of adverse outcomes, to allow for more targeted treatment strategies.

The advent of non-invasive methods such as transient elastography (TE) (Fibroscan, Paris) to detect hepatic steatosis and liver fibrosis has brought better understanding to the entity of NAFLD, particularly among adults. TE reliability and consistency on the assessment of liver fibrosis compared to gold standard liver biopsy has been proven in adult population (6, 7). Currently, there is no consensus in usage of TE among pediatric population, with several studies showing TE is accurate in identifying hepatic steatosis and fibrosis compared to other non-invasive assessments such as ultrasound (8–10). Although liver biopsy remains the reference standard for assessment of the degree of steatosis, its invasiveness, cost, and risk of complications are less tolerable in children and adolescents than in adults (11). Therefore, utilizing this non-invasive reproducible method of assessment, particularly among high risk groups, allows better screening of NAFLD in children.

In this study, we aim to investigate the relationship between hepatic steatosis, biochemical parameters of liver profile and components of the metabolic syndrome with severity of liver fibrosis among children with obesity and diabetes mellitus.

## Methods

### Study Population

We conducted a cross-sectional study involving 57 consecutive children with age range of 6–18 years old from pediatric obesity and diabetes clinic in University Malaya Medical Center (UMMC) from 2016 to 2019. These children underwent liver stiffness measurement (LSM) and controlled attenuation parameter (CAP) using transient elastography (FibroScan, Echosens, Paris). Written informed consent was obtained from parent/legal guardian of children under 18 years of age, 18 years old adolescent provided their own consent. Participants with viral hepatitis A, B, or C infection, autoimmune hepatitis, history of alcohol intake, drug-induced fatty liver, genetic disorders, or any other causes of chronic liver diseases were excluded from the study.

### Clinical and Laboratory Variables

Demographic data such as age, gender, and ethnicity were included. Anthropometric measurements including body weight, height and waist circumference (WC), and blood pressure (BP) were measured and recorded using standard equipment. BMI was calculated according to the following formula: BMI = weight in kg/height in meters^2^. BMI-for-age percentile was calculated using World Health Organization (WHO) growth chart and was classified according to the following; >+1 standard deviation (SD) as overweight, >+2 SD as obesity, and >+3 SD as morbid obesity (12).

Laboratory variables such as liver function test and biochemistry profile [alanine aminotransferase (ALT), aspartate aminotransferase (AST)], gamma-glutamyl transferase [GGT] and baseline platelet count were collected at the time point of NAFLD diagnosis. ALT > 25 IU/L was considered abnormal in boys and >22 IU/L in girls (13). Other metabolic components such as history of diabetes mellitus status, fasting blood glucose, hemoglobin A1c (HbA1c), fasting serum insulin, history of hypertension status, systolic and diastolic blood pressure, history of dyslipidemia status, and lipid profile [total cholesterol (TC), serum triglycerides (TG) level, serum high-density lipoprotein (HDL) cholesterol level, serum low-density lipoprotein (LDL) cholesterol level] were also collected. Homeostatic Model Assessment for Insulin Resistance (HOMA-IR) model was used to quantify insulin resistance with the formula: [fasting plasma glucose (mmol/l) × fasting serum insulin (mU/l)]/22.5 (14, 15). High HOMA-IR value imply low insulin sensitivity and vice versa (14). HOMA-IR value of >4.0 indicates insulin resistance (16–18).

Metabolic syndrome was defined using the International Diabetes Federation (IDF) criteria with presence of abdominal obesity (WC ≥ 90th percentile) and 2 or more of the following criteria: serum triglyceride (TG) level ≥ 1.7 mmol/L, serum HDL-cholesterol <1.03 mmol/L, systolic blood pressure (SBP) of ≥130 mmHg or diastolic blood pressure (DBP) of ≥85 mmHg and fasting blood glucose (FBG) ≥5.6 mmol/L or known type 2 diabetes mellitus (T2DM) (19). Children younger than 10 years old who fulfilled the above criteria, are considered as at risk of metabolic syndrome (19).

### Non-invasive Assessment of Steatosis and Fibrosis

Transient elastography (TE) (FibroScan, Echosens, Paris) was performed by a dedicated and specially trained personnel using the 3.5-MHz M probe to obtain CAP and LSM score. We stratified patients according to the cut-offs for CAP scores as ≤ 248 dB/m (normal), 249–268 dB/m (mild steatosis), 269–280 dB/m (moderate steatosis), and >280 dB/m (severe steatosis) (8). Moreover, we considered the cut-offs for LSM score as <7.0 kPA (F0–1, no significant fibrosis), 7.0–8.6 kPa (F ≥ 2, mild fibrosis), 8.7–10.2 kPa (F ≥ 3, moderate fibrosis), and ≥10.3 kPa (F4, cirrhosis) (9).

The Pediatric NAFLD Fibrosis Score (PNFS) was calculated using serum ALT, ALP, GGT levels, and platelet counts (20). The following formula was used: z = 1.1 + (0.34 ^*^ SQRT(ALT)) + (0.002^*^ALP) – (1.1^*^log(platelets) – (0.02^*^GGT)) (20). A probability distribution (p) with a value between 0 and 100 was then calculated by the following formula: *p* = 100 × exp(z)/[1 + exp(z)] (20).

### Statistical Analysis

We calculated our sample size based on difference in means of CAP and LSM between obese children and non-obese children. The effect size (Cohen d) for CAP and LSM is 2.06 and 0.92, respectively, giving a sample size of 16 and 32, respectively. Our study recruited 57 patients in anticipation of drop-out.

Data analysis were carried out using SPSS version 23.0 (Chicago, IL, USA). Values were presented as mean ± standard deviation or median and interquartile range (IQR) for continuous variables while categorical variables were recorded as number and percentages. All variables were tested for normality using Kolmogorov-Smirnov test. Student's *t*-test was used for normally distributed variables whereas Mann-Whitney *U*-test was used for skewed variables. Correlations were assessed using Spearman's correlation coefficient. Univariate and multivariate analyses were performed to identify factors associated with progression of liver fibrosis. Receiver operating characteristic (ROC) curve analysis was used to identify the cut-off value of the PNFS with the highest sensitivity and specificity. A two-tailed *p*-value significance level of 0.05 was used for all test performed.

## Results

The demographic data and clinical characteristics of all subjects are summarized in Table 1. Among 57 subjects, 34 (60%) were males and 23 (40%) were females. More than half of our study population constitutes children of Malay ethnicity (64.9%). The median age of the study population was 13 years old with age ranging from 6 to 18 years' old. Among 44 (77.2%) patients with steatosis, 40 (70.2%) had severe steatosis. Liver fibrosis of stage F ≥ 2 was detected in 18 (40.9%) children with hepatic steatosis, of which 10 (22.7%) had fibrosis stage 2, 4 (9.1%) had fibrosis stage 3 and 4 (9.1%) children had liver cirrhosis or fibrosis stage 4. There were 18 (31.6%) who had diabetes mellitus (DM), 5 (8.8%) had hypertension, and 23 (40.4%) fulfilled the criteria for metabolic syndrome. Four out of eight of the patients with advanced liver fibrosis or cirrhosis had metabolic syndrome. According to WHO BMI-for-age percentile, 50 (87.7%) of our study population are obese, of which, 27 (54%) of them are morbidly obese.

**Table 1:** Demographic and clinical characteristics of overweight and obese children (*n* = 57).

**Variable**	**All patients (*n* = 57)**
**Gender**, male (%)	34 (59.6)
**Ethnicity**, *n* (%)	
Malay	37 (64.9)
Chinese	7 (12.3)
Indian	11 (19.3)
Others	2 (3.5)
**Age**, years	13 (11,15)
**CAP score** (dB/m)	300 (263, 329)
**Severity of steatosis**, *n* (%)	
Normal	13 (22.8)
Mild	2 (3.5)
Moderate	2 (3.5)
Severe	40 (70.2)
**LSM score** (kPa)	6.3 (3.9, 8.1)
**Severity of fibrosis (overall)**, *n* (%)	
No significant fibrosis (F0–1)	37 (64.9)
Mild (F2)	10 (17.5)
Moderate (F3)	5 (8.8)
Severe (F4)	5 (8.8)
**Severity of fibrosis (among patients with steatosis)**, *n* (%)	
No significant fibrosis (F0–1)	26 (59.1)
Mild (F2)	10 (22.7)
Moderate (F3)	4 (9.1)
Severe (F4)	4 (9.1)
**Severity of fibrosis (among patients with metabolic syndrome)**, *n* (%)	
No significant fibrosis (F0–1)	11 (47.8)
Mild (F2)	7 (30.4)
Moderate (F3)	3 (13.0)
Severe (F4)	2 (8.7)
**ALT** (UL^−1^)	28.0 (18.0, 55.5)
**AST** (UL^−1^)	26.0 (19.0, 35.0)
**GGT** (UL^−1^)	24.0 (17.0, 44.0)
**Platelet** (10^9^ L^−1^)	344 (270, 381)
**Diabetes Mellitus (DM)**, *n* (%)	18 (31.6)
**Fasting blood sugar (FBS)** (mmol L^−1^)	4.8 (4.6, 5.4)
**HbA1c** (%)	5.5 (5.3, 6.0)
**Fasting serum insulin** (mU/L)	24.2 (17.7, 35.4)
**HOMA-IR**	5.0 (3.6, 8.3)
**Hypertension**, *n* (%)	5 (8.8)
**Blood pressure**	
Systolic (mmHg)	118 (114, 125)
Diastolic (mmHg)	66 (62,73)
**Triglyceride (TG)** (mmol L^−1^)	1.4 (1.0, 2.1)
**Total cholesterol** (mmol L^−1^)	4.6 (3.9, 5.0)
**HDL-C** (mmol L^−1^)	1.10 (0.93, 1.20)
**LDL-C** (mmol L^−1^)	2.78 (2.32, 3.14)
**TG: HDL-C ratio**	1.3 (0.9, 2.1)
**BMI** (kg m^−2^)	29.8 (27.1, 33.2)
**BMI category**, *n* (%)	
Normal	1 (1.8)
Overweight	6 (10.5)
Obese	23 (40.4)
Morbid obese	27 (47.4)
**Waist circumference** (cm)	92.0 (84.5, 101.3)
Male	97.0 (86.6, 107.1)
Female	88.0 (79.0, 92.5)
**Metabolic syndrome**, *n* (%)	23 (40.4)

*All data are expressed as median (interquartile range) unless specified. CAP, controlled attenuation parameter; LSM, liver stiffness measurement; ALT, alanine aminotransferase; AST, aspartate aminotransferase; GGT, gamma-glutamyl transferase; HOMA-IR, Homeostatic Model Assessment for Insulin Resistance; HDL-C, high-density lipoprotein cholesterol; LDL-C, low-density lipoprotein cholesterol; TG:HDL-C ratio, triglyceride to high-density lipoprotein cholesterol ratio; BMI, body mass index*.

Clinical characteristics were compared between patients with hepatic steatosis and liver fibrosis as shown in Table 2. Patients with F ≥ 2 liver fibrosis are categorized as presence of fibrosis. Our analyses showed that BMI were significantly higher in patients with steatosis than in patients without steatosis (*p* = 0.003). Metabolic components such as HbA1c, SBP, serum TG levels, serum TC level, triglyceride to high density lipoprotein cholesterol (TG:HDL-C) ratio, BMI, and WC were significantly higher in patients with fibrosis (*p* < 0.05). DM and HOMA-IR were also significantly associated with fibrosis (*p* < 0.05). Fibrosis is three times more likely to occur in the presence of metabolic syndrome (OR = 3.545, 95% CI: 1.135–11.075, *p* = 0.026). Moreover, serum ALT, AST, and GGT levels were significantly higher in patients with steatosis and F ≥ 2 fibrosis (*p* < 0.05).

**Table 2:** Comparison of demographic, clinical characteristics, and metabolic factors between children and adolescents with or without liver steatosis and with or without liver fibrosis.

**Variable**	**Steatosis (*n* = 44)**	**Without steatosis (*n* = 13)**	***P* value**	**Fibrosis (*n* = 20)**	**Without fibrosis (*n* = 37)**	***P* value**
**Gender**, male (%)	26 (59.1)	8 (61.5)	0.874	12 (60.0)	22 (59.5)	0.968
**Ethnicity**, *n* (%)			0.032^*^			0.248
Malay	32 (72.7)	5 (38.5)		15 (75.0)	22 (59.5)	
Chinese	5 (11.4)	2 (15.4)		3 (15.0)	4 (10.8)	
Indian	5 (11.4)	6 (46.2)		1 (5.0)	10 (27.0)	
Others	2 (4.5)	0		1 (5.0)	1 (2.7)	
**Age**, years	13 (11,15)	13 (11,15)	0.632	14 (12,16)	12 (10,14)	0.007^**^
**CAP score** (dB/m)	315 (293, 346)	200 (133, 229)	0.0001^***^	306 (288, 350)	300 (231, 325)	0.195
**Severity of steatosis**, *n* (%)			–			0.387
Normal	–	13 (100)		2 (10.0)	11 (29.7)	
Mild	2 (4.5)	–		1 (5.0)	1 (2.7)	
Moderate	2 (4.5)	–		1 (5.0)	1 (2.7)	
Severe	40 (90.9)	–		16 (80.0)	24 (64.9)	
**LSM score** (kPa)	6.6 (4.1, 8.4)	4.7 (3.6, 6.5)	0.156	8.95 (7.65, 11.05)	4.3 (3.6, 6.1)	0.0001^***^
**Severity of fibrosis**, *n* (%)			0.261			–
No significant fibrosis	26 (59.1)	11 (84.6)		–	37 (100)	
Mild	10 (22.7)	0		10 (50.0)	–	
Moderate	4 (9.1)	1 (7.7)		5 (25.0)	–	
Severe	4 (9.1)	1 (7.7)		5 (25.0)	–	
**ALT** (UL^−1^)	32.5 (22.3, 80.3)	18.0 (13.5, 32.5)	0.003^**^	73.0 (38.8, 101.5)	23.0 (15.5, 32.0)	<0.0001^***^
**AST** (UL^−1^)	28.0 (22.0, 39.0)	18.0 (15.8, 22.5)	0.012^*^	37.0 (23.0, 60.0)	23.0 (17.0, 28.5)	0.011^*^
**GGT** (UL^−1^)	26.0 (18.3, 59.3)	17.0 (12.5, 26.0)	0.007^**^	57.5 (31.0, 72.0)	18.0 (15.0, 25.5)	<0.0001^***^
**Platelet** (10^9^ L^−1^)	357 (302, 395)	275 (236, 333)	0.059	314 (246, 368)	354 (281, 405)	0.175
**Diabetes Mellitus (DM)**, *n* (%)	13 (29.5)	5 (38.5)	0.543	10 (50.0)	8 (21.6)	0.028^*^
**Fasting blood sugar (FBS)** (mmol L^−1^)	4.9 (4.6, 5.6)	4.8 (4.6, 4.9)	0.370	5.1 (4.5, 8.8)	4.8 (4.6, 5.1)	0.241
**HbA1c** (%)	5.5 (5.2, 6.0)	5.4 (5.1, 6.3)	0.696	5.7 (5.4, 7.6)	5.4 (5.2, 5.7)	0.035^*^
**Fasting serum insulin** (mU/L)	24.2 (16.8, 37.1)	23.0 (17.2, 41.8)	0.969	24.1 (19.3, 33.9)	27.1 (15.2, 38.8)	0.814
**HOMA-IR**	5.1 (3.8, 9.1)	4.5 (1.9, 9.3)	0.404	7.1 (5.3, 13.0)	4.2 (3.0, 6.8)	0.005^**^
**Hypertension**, *n* (%)	4 (9.1)	1 (7.7)	0.876	1 (5.0)	4 (10.8)	0.459
**Blood pressure**						
Systolic (mmHg)	118 (112, 124)	122 (114, 128)	0.589	121 (116, 131)	116 (109, 123)	0.039^*^
Diastolic (mmHg)	65 (63,73)	67 (57,71)	0.514	67 (64,74)	65 (62,73)	0.348
**Triglyceride (TG)** (mmol L^−1^)	1.5 (1.0, 2.2)	1.3 (0.85, 1.85)	0.418	2.05 (1.1, 2.3)	1.3 (0.8, 1.7)	0.005^**^
**Total cholesterol** (mmol L^−1^)	4.7 (4.0, 5.2)	4.3 (3.6, 4.7)	0.078	5.0 (4.1, 5.8)	4.4 (3.8, 4.8)	0.024^*^
**HDL-C** (mmol L^−1^)	1.1 (0.9, 1.2)	1.13 (1.04, 1.16)	0.524	1.1 (0.9, 1.2)	1.10 (0.9, 1.2)	0.482
**LDL-C**(mmol L^−1^)	2.97 (2.42, 3.26)	2.54 (2.16, 2.94)	0.092	2.97 (2.51, 3.83)	2.64 (2.26, 3.03)	0.141
**TG: HDL-C ratio**	1.3 (0.9, 2.2)	1.1 (0.9, 1.7)	0.318	1.9 (1.0, 2.4)	1.2 (0.8, 1.4)	0.012^*^
**BMI** (kg m^−2^)	30.5 (27.9, 34.3)	26.4 (22.2, 29.6)	0.003^**^	33.5 (27.9, 37.8)	29.5 (25.6, 31.9)	0.009^**^
**BMI category**, *n* (%)			0.001^**^			0.493
Normal	0	1 (7.7)		0	1 (2.7)	
Overweight	2 (4.5)	4 (30.8)		2 (10.0)	4 (10.8)	
Obese	16 (36.4)	7 (53.8)		6 (30.0)	17 (45.9)	
Morbid obese	26 (59.1)	1 (7.7)		12 (60.0)	15 (40.5)	
**Waist circumference** (cm)	94.5 (86.0, 104.5)	85.8 (73.0, 95.4)	0.059	99.2 (91.5, 114.8)	89.0 (83.5, 97.5)	0.005^**^
Male	97.5 (90.5, 107.1)	90.3 (84.6, 106.9)	0.191	106.3 (97.0, 116.3)	91.0 (84.6, 98.8)	0.001^**^
Female	89.0 (82.5, 94.5)	71.0 (66.1, 85.3)	0.035^*^	88.0 (81.6, 100.0)	88.0 (71.0, 93.5)	0.598
**Metabolic syndrome**, *n* (%)	20 (45.5)	3 (23.1)	0.148	12 (60.0)	11 (29.7)	0.026^*^

*All data are expressed as median (interquartile range) unless specified. P values are calculated by Chi- square test for categorical variable and Mann-Whitney U-test for continuous variables. P < 0.05 (statistically significant). CAP, controlled attenuation parameter; LSM, liver stiffness measurement; ALT, alanine aminotransferase; AST, aspartate aminotransferase; GGT, gamma-glutamyl transferase; HOMA-IR, Homeostatic Model Assessment for Insulin Resistance; HDL-C, high-density lipoprotein cholesterol; LDL-C, low-density lipoprotein cholesterol; TG:HDL-C ratio, triglyceride to high-density lipoprotein cholesterol ratio; BMI, body mass index. Bold values represent statistically significant values*.

* P < 0.05,

** P < 0.01,

*** *P < 0.001*.

### Metabolic Predictors of Liver Fibrosis

Multivariate regression analysis was performed to identify metabolic components which are independently associated with liver fibrosis (Table 3). The overall significance of the model was *p* < 0.001 after adjusting for age. The only significant independent predictor of liver fibrosis is waist circumference. For every increase in 1 cm of waist circumference, LSM value increases by 0.1 kPa (95% CI: 0.036, 0.168; *p* = 0.003). TG:HDL-C ratio, HOMA-IR, and hypertension were not independent predictors of liver fibrosis.

**Table 3:** Metabolic components independently associated with liver fibrosis (LSM value) in overweight and obese children and adolescents.

**Metabolic components**	**LSM value**			
	**Unadjusted for age**		**Adjusted for age**	
	**CV (95% CI)**	***P* value**	**CV (95% CI)**	***P* value**
Waist circumference	**0.125 (0.069, 0.181)**	0.0001^***^	**0.102 (0.036, 0.168)**	0.003^**^
TG:HDL-C ratio	−0.075 (−0.982, 0.832)	0.868	0.004 (−0.902, 0.911)	0.993
HOMA-IR	−0.008 (−0.074, 0.057)	0.797	−0.015 (−0.081, 0.050)	0.642
Hypertension	−1.128 (−3.610, 1.353)	0.363	−1.187 (−3.647, 1.273)	0.335

*P < 0.05 (statistically significant). TG:HDL-C ratio, triglyceride to high-density lipoprotein cholesterol ratio; HOMA-IR, Homeostatic Model Assessment for Insulin Resistance. Bold values represent statistically significant values*.

** P < 0.01,

*** *P < 0.001*.

### Use of Pediatric NAFLD Fibrosis Score (PNFS)

**Figure 1:**
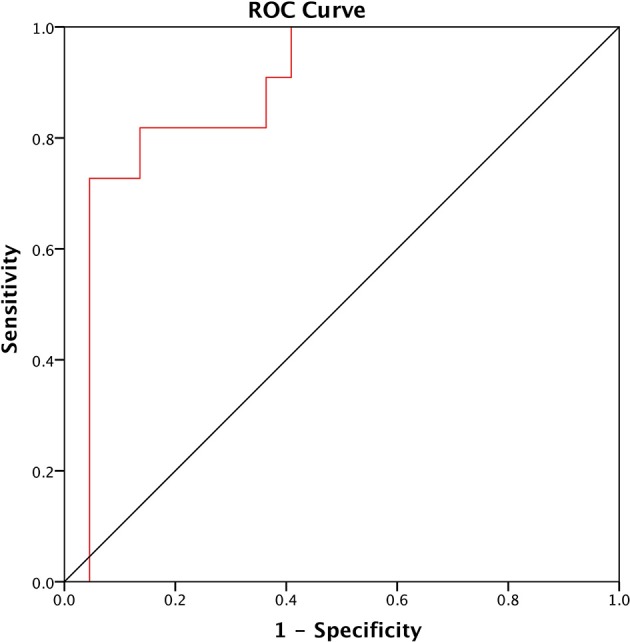
ROC of the Pediatric NAFLD Fibrosis Score (PNFS) to distinguish between NAFLD patients with and without fibrosis. The area under the ROC curve (AUROC) is 0.884 (95% CI: 0.763, 1.00; *p* < 0.0001).

PNFS identified patients with fibrosis with an area under the ROC curve of 0.884 (95% CI: 0.763, 1.00; *p* < 0.0001) (Figure 1). A cut-off value of 76.6 and above detected the presence of fibrosis with a sensitivity of 81.8% and a specificity of 86.4% (positive predictive value (PPV) of 81.8% and negative predictive value (NPV) of 86.4%).

## Discussion

NAFLD is prevalent among children with diabetes or obesity in Malaysia with more than 75% developed hepatic steatosis and 41% developed liver fibrosis of stage 2 or above among those with steatosis. More worryingly, of those who had hepatic steatosis, almost half of the study population had significant or advanced fibrosis. Our prevalence of NAFLD among obese or diabetic children is notably higher compared to the estimated pooled prevalence of NAFLD in children, which is reported to be around 34.2% (95% CI: 27.8–41.2%) globally and 62.3% (95% CI: 34.9–83.6%) in Asia (2). Asians develop obesity and diabetes-related complications at a lower BMI than Western counterparts due to the higher deposition of visceral fat among Asian despite having similar BMI with whites (21–27). Although these landmark studies were carried out primarily in adults, similar observations were made in pediatric population, in which Asian children have increased body fat in comparison to White children at the same body mass index (28–31). For example, a national cross-sectional study done among primary schoolchildren in United Kingdom found that UK South Asian children have higher adiposity levels compared with white Europeans, but these differences were not well-represented by comparisons based on BMI, which systematically underestimates adiposity in South Asians because of its association with height (31). After BMI adjustments using regression model, South Asian children in England were revealed to have extremely high overweight-obesity prevalence (29). This further supports the theory that Asians may develop obesity-related complications at a lower BMI. We highlight that in our study, median BMI was 29.8 kg/m^2^ with close to 90% being obese or morbidly obese. This could also account for the high prevalence of fatty liver detected, as prevalence of NAFLD increased considerably on average with increasing BMI category, keeping in mind that children may already develop fatty liver even at a lower BMI in view of increased adiposity (2). Moreover, even among children who had similar environmental exposure, there is inter-ethnic differences in hepatic fat accumulation with Malay predominantly affected. This has been demonstrated by local studies in multi-ethnics Malaysia (32, 33), in which the prevalence of NAFLD were consistently higher among the Malays compared to Indians and Chinese, and this ethnic predilection could be observed as early as young adulthood (34).

In this study, metabolic syndrome was found to be significantly associated with presence of significant or advanced liver fibrosis, consistent with previous studies (35, 36). Patton et al.'s study on 254 biopsy-proven NAFLD children has proven that metabolic syndrome, apart from being predictive of steatosis severity, is significantly associated with advanced liver fibrosis (35). Presence of one or more metabolic syndrome criteria was also demonstrated to augment the risk of liver fibrosis in another similar study, providing us the potential of utilizing metabolic syndrome as a prognostic indicator of severity of liver disease (36). Hence, we decided to identify the independent predictors of fibrosis among metabolic components in this study. Multiple regression further reveals that WC was the only independent predictor for liver fibrosis among metabolic components such as HOMA-IR, TG:HDL-C ratio and hypertension. Several studies in children population have shown that WC is a significant risk factor for liver fibrosis and it could predict risk of NAFLD in obese children (37, 38). This suggests that abdominal rather than generalized obesity has a greater contribution to liver fibrosis (38). Abdominal obesity leads to reduction of hepatic and insulin sensitivity (39), as visceral adipose tissue is highly resistant to insulin and susceptible to lipolysis, thus producing excess free fatty acids than adipose tissue in other sites (40). The greater availability of substrate for lipogenesis, combined with the relative hyperinsulinemia effect further enhances hepatic lipogenesis, leading to a vicious cycle (41). Moreover, growing evidence suggest that obesity leads to activation of various inflammatory pathways with increased pro-inflammatory interleukins and cytokines activity (42–45). This supports the two-hit model theory of liver fibrosis proposed by Buzzetti et al., who suggested that the first hit consisted of fat accumulation in the liver, high fat diet, obesity, insulin resistance, and sedentary lifestyle, with the second hit consisted of activation of inflammatory process leading to fibrogenesis (46). Though WC does not accurately reflect the visceral/abdominal adiposity due to gender and ethnic differences (47, 48), its reliability could be improved further when combined with height or hip circumference, such as waist-circumference height ratio or waist-hip ratio (49–52). Thus, WC could be a powerful marker for NAFLD and should be utilized more frequently among obese children, as advanced non-invasive imaging such as transient elastography may not be universally available and the invasiveness of liver biopsy preclude its usage except in direst of cases. Unlike several studies which reported HOMA-IR and its surrogate marker, TG:HDL-C ratio as an independent predictor of NAFLD, insulin resistance is not an independent predictor of liver fibrosis in our study (53–56). However, there is a significant association of insulin resistance and TG:HDL-C ratio with liver fibrosis in the presence of other metabolic parameters, explaining its pivotal role in the pathogenesis of NAFLD (57, 58).

According to a national cohort study with 39 years follow-up, overweight in late adolescents was a risk factor for developing severe liver disease later in life, and development of T2DM during follow-up was independently associated with a further increased risk of severe liver disease (5, 59). In our study, among children with metabolic syndrome, more than half of them has F ≥ 2 liver fibrosis. Intensive treatment of their underlying metabolic diseases such as lifestyle modification and tailored pharmacological management is essential to prevent further progression of liver damage, which could have disastrous consequences in future. Ideally, all pediatric patients with obesity or DM should be managed by dedicated pediatric endocrinology teams with specialist diabetes nurse, dietitian, psychologist, and sports physicians' input. However, this multidisciplinary approach may not be widely practiced, and in the case of middle-income countries, such unit would only be available in major cities with limited human resources. As such, we need to prioritize patients who needs close supervision and intensive treatment strategies.

Despite the huge burden of hepatic steatosis in children, unexpectedly we did not find any relationship between hepatic steatosis and liver fibrosis in our study. However, metabolic components such as HbA1c, HOMA-IR, SBP, serum TG level, serum TC level, TG:HDL-C ratio, BMI, and WC are significantly associated with liver fibrosis. Contrary to our study, a hospital-based study in Japan found that LSM was highly correlated with CAP among obese group (60). In spite that most of our populations were obese (87.7%), we did not find similar correlations. Given the natural history of NAFLD, which has low annual incidence of fibrosis progression, we postulate that the development of fibrosis in this cohort of patient is likely contributed by the inflammatory response induced by metabolic components rather than liver steatosis itself due to relatively short exposure duration (61–64).

Multiple models have been developed to predict risk of fibrosis (20, 65–68), and we evaluated PNFS as a predictive tool for liver fibrosis. PNFS is an excellent prediction tool for liver fibrosis among children with an AUROC of 0.884, comparable with other studies with AUROC of 0.74 and 0.85, respectively (20, 68). Alkhouri et al. reported that PNFS was more accurate for predicting advanced fibrosis than APRI, FIB-4 index and NAFLD fibrosis score (20). One important advantage of PNFS is utilization of values easily obtained from liver function test (ALP, ALT, GGT) and full blood count (platelet). Thus, PNFS could be use in both primary care or pediatrics setting to identify patients with probable liver fibrosis for further investigation and management.

Our study is the first study in South East Asia looking into the relationship between metabolic syndrome and liver fibrosis in diabetic or obese pediatric population by utilizing transient elastography to assess hepatic steatosis and liver fibrosis. The data were prospectively collected from a cross-sectional study, hence minimizing potential inaccuracy or incomplete data. Furthermore, our hospital-based study is conducted in a multi-ethnic population with similar environmental exposure. One of the limitations in our study is a relatively small population size, but adequately powered for statistical significance. In addition, liver biopsy, the gold standard for diagnosing NAFLD, was not performed in our study subjects as it is invasive with potential complication risks. MRI was not used as our imaging modality because of its high cost. Liver ultrasound elastography is suitable as non-invasive assessment tool. There are different techniques available within liver ultrasonography, such as vibration-controlled transient elastography (TE), two-dimensional shear-wave elastography (2D-SWE) and point shear-wave elastography (pSWE) (69). These methods are broadly similar, but differ in terms of technical principles and sampling rate (70). Although these methods correlate with each other, specific results are not easily convertible interchangeably (71, 72). The major confounding factors were severe inflammation, cholestasis, liver congestion, or presence of other liver disorders (69, 70, 72). Therefore, children with any other concomitant liver disease were excluded from our study to prevent overestimation of liver fibrosis while performing LSM with TE. Sensitivity analysis conducted as recommended by Karlas et al. and Petta et al. improve study reliability as it accounts for severity of steatosis, presence of diabetes mellitus, and body mass index (73, 74). Guidelines in relation to non-invasive evaluation of liver disease were developed in adult population and should be validated in children (70, 75). According to a meta-analysis, both TE and pSWE showed similar accuracy in staging liver fibrosis among NAFLD patients, but no direct comparisons were made (76). In contrast, a recent head-to-head comparison in NAFLD adult cohort, showed that TE was significantly better than pSWE for the diagnosis of moderate to advanced liver fibrosis (77). Meanwhile, another study comparing between TE, 2D-SWE, and pSWE in healthy children showed excellent feasibility for all methods, albeit 2D-SWE showed significantly lower LSM values than pSWE and TE, raising concern of underestimating liver fibrosis in children (78). SWE provides anatomical representation with real-time image acquisition and uses existing ultrasound B mode, allowing complete assessment in a single setting, thus reducing costs (79). Further studies are needed to compare the efficacy of TE and SWE in both adult and children NAFLD cohort.

In conclusion, obese children with metabolic syndrome are more likely to have advanced liver fibrosis and WC predicts risk of liver fibrosis among children with NAFLD. Hence, we advocate that waist circumference should be obtained, combining risk score such as PNFS to better select patients most at risk of advanced liver disease. As fibrosis is the most important predictor of adverse outcome in NAFLD, presence of advanced liver fibrosis at young age may increase risk of earlier onset of liver-related morbidity and mortality.

## Data Availability Statement

The datasets generated for this study are available on request to the corresponding author.

## Ethics Statement

The studies involving human participants were reviewed and approved by University Malaya Medical Center (UMMC) Medical Research Ethics Committee (MREC). Written informed consent was obtained from parent/legal guardian of children under 18 years of age.

## Author Contributions

RM, AA, and MJ designed the research study. Y-WT, AA, and MJ established cohort and collected the clinical data for patients. Y-WT carried out data analysis and documented the findings. Y-WT and S-WW wrote the manuscript. RM, AA, MJ, and S-WW provided critical inputs to the manuscripts. All authors proof read the manuscript.

### Conflict of Interest

The authors declare that the research was conducted in the absence of any commercial or financial relationships that could be construed as a potential conflict of interest.
